# Cysticercosis and neurocysticercosis in people from Mocuba district, Zambézia province: A Mozambican community-based study

**DOI:** 10.1371/journal.pntd.0013083

**Published:** 2025-05-13

**Authors:** Janny Mucavele, Noémia Nhancupe, Gaby Ermelindo Roberto Monteiro, Regina Daniel Miambo, Lídia Gouveia, Alberto Pondja, Irina Mendes Sousa, Stephen W. Bickler, Constance A. Benson, Robert T. Schooley, Veronika Schmidt, Charlotte Ruether, Dominik Stelzle, Clarissa Prazeres da Costa, Ladino Suade, Milton L. Wainberg, Maria A. Oquendo, Andrea S. Winkler, Emilia Virginia Noormahomed, Zambézia cysticercosis working Group

**Affiliations:** 1 Department of Mental Health, Matola Provincial Hospital, Matola, Mozambique; 2 Mozambique Institute for Health Education and Research (MIHER), Maputo, Mozambique; 3 Department of Microbiology, Faculty of Medicine, Eduardo Mondlane University, Maputo, Mozambique; 4 Department of Animal and Public Health, Faculty of Veterinary Medicine, Eduardo Mondlane University, Maputo, Mozambique; 5 Department of Mental Health, National Public Health Directorate, Mozambique Ministry of Health, Maputo, Mozambique; 6 Faculty of Sciences, Department of Biological Sciences, Eduardo Mondlane University, Maputo, Mozambique; 7 Department of Surgery, Division of Pediatric Surgery, University of California San Diego School of Medicine, San Diego, California, United States of America; 8 Department of Medicine, Division of Infectious Diseases and Global Public Health, School of Medicine, University of California, San Diego, California, United States of America; 9 Center for Global Health, School of Medicine and Health, Technical University of Munich, Munich, Germany; 10 Department of Neurology, TUM, Munich, Germany; 11 Department of Neuroradiology, RoMed Hospital Rosenheim, Munich, German; 12 Institute for Medical Microbiology, Immunology and Hygiene, School of Medicine and Health, Technical University of Munich, Munich, German; 13 Department of Surgery, Quelimane Central Hospital, Quelimane, Mozambique; 14 Department of Psychiatry, New York State Psychiatric Institute, New York, New York, United States of America; 15 Columbia University College of Psysichians and Surgeons, New York, New York, United States of America; 16 Department of Psychiatry, Perelman School of Medicine, University of Pennsylvania, Philadelphia, Pensylvania, United States of America; 17 Department of Community Medicine and Global Health, Institute of Health and Society, University of Oslo, Oslo, Norway; KEMRI-Wellcome Trust Research Programme: Centre for Geographic Medicine Research Coast, KENYA

## Abstract

**Introduction:**

*Taenia solium* cysticercosis represents a significant public health concern, especially in low-income countries such as Mozambique, where especially the sub form neurocysticercosis can be associated with acute symptomatic seizures, epilepsy and other neurological and psychiatric disorders. Therefore, this study aimed to determine the prevalence of *T. solium* cysticercosis, neurocysticercosis, seizures and chronic headaches in the Mocuba district, Zambézia province, Mozambique, and to assess their associations.

**Methods:**

The overall study combined both a community-based and a clinic-based segment, including epidemiological, clinical, laboratory and neuroradiological approaches, to investigate the prevalence and the association of cysticercosis, neurocysticercosis, seizure activity and chronic headaches in the Mocuba district. The community-based study involved 6,932 participants who were asked a questionnaire related to signs/symptoms of neurocysticercosis and who were asked to provide a blood sample for serological testing. Serological tests (Antigen-ELISA and Western blot) were used to detect cysticerci specific antigens and antibodies, respectively, in the participants. The clinic-based study included cerebral computed tomography (CT) of 233 individuals – a subset of those recruited from the community (with and without seizure activity and with and without cysticercosis based on serology).

**Results:**

The prevalence of seizures and chronic headaches in the community-based study was 6.5% and 46.2% respectively, and the cysticercosis seroprevalence was 9.6%. Seizures and chronic headaches presented significant associations with cysticercosis seropositivity (p < 0.05). The clinic-based study revealed 9 (3.9%) of 233 individuals with neurocysticercosis-typical lesions on CT-scan of whom one case was negative on serology and on screening for seizure activity.

**Conclusions:**

The community-based prevalence of seizure activity and cysticercosis was high in the Mocuba district. There was also a significant association of seizure activity and chronic headaches with the seroprevalence of cysticercosis and 8 out of 9 people with neurocysticercosis had seizure activity. This highlights the importance of increased awareness and the need for building health literacy within the healthcare workforce and the communities as well as the implementation of targeted interventions, both for people with seizure activity with and without neurocysticercosis.

Future research should also assess the impact of preventive measures in reducing disease burden caused by *T. solium*.

## Author summary

Neurocysticercosis, a parasitic neglected tropical disease caused by the larva of *Taenia solium* poses a substantial public health problem in low-income countries where it strongly overlaps with epilepsy. Mozambique is no exception. A recent pilot epidemiological study in the Mocuba district, Zambézia Province, central Mozambique, reported a seroprevalence of human cysticercosis of 10.3%. Despite those results and significant efforts to diagnosis and control neurocysticercosis, which relies on a combination of neuroimaging and serological tools, that are hardly available for most of people in low-income countries, the prevalence of cysticercosis and neurocysticercosis and its association with neurological conditions, such as seizures and chronic headaches, so far has not been investigated in large-scale studies in Mozambique.

In this community-based and clinic-based study that included 6932 people, we sought to determine the prevalence of cysticercosis and neurocysticercosis, as well as its possible association with seizure activity and chronic headaches in the Mocuba district, Zambézia province. Our study revealed that the prevalence of seizure activity and chronic headaches in the community-based study was 6.5% and 46.2% respectively, and that the cysticercosis seroprevalence was 9.6%. Seizures and chronic headaches were significantly associated with cysticercosis seropositivity (p < 0.05). The clinic-based study, that included 233 individuals from the community-based arm of the study, revealed that 9 (3.9%) of study participants had neurocysticercosis and of those one screened negative for cysticercosis on serology and negative for seizure activity.

We hope that our findings will provide valuable insights for key stakeholders and policymakers to take appropriate actions to be able to effectively diagnose and manage clinical cases of epileptic seizures/epilepsy, chronic headache and *T. solium* cysticercosis/neurocysticercosis and to develop/adjust public health measures for control and elimination of the respective diseases cause by *T. solium.*

## Introduction

*Taenia solium* cysticercosis (CC), caused by the larva of *T. solium*, is a neglected zoonotic disease primarily affecting humans as definitive (= taeniasis) and pigs as intermediate hosts (= porcine CC) [1,2]. However, man can accidentally ingest eggs of *T. solium* mainly through water or contaminated food and act as intermediate host. Once the eggs are ingested, they hatch, and the larva migrates to different parts of the body including muscles, eye and central nervous system. The most serious clinical presentation are the ones related to the presence of the parasite in the human central nervous system; this disease is called neurocysticercosis (NCC) [1,3]. The neurological signs and symptoms of NCC are dependent on the number and location of the parasite, parenchymal (in the brain and the spinal cord) or extraparenchymal (in the ventricular and subarachnoid spaces), and on the larval stage of the parasite which includes vesicular, colloidal, granular and calcified forms [4,5].

The immunological pathomechanisms contributing to the clinical presentation of NCC are related to inflammatory reactions at both local and systemic levels and are dependent on the stage of the parasite [6]. Generally, in the vesicular stage affected individuals remain asymptomatic due to immunological tolerance established between the host and the parasite. The colloidal stage, which occurs when the parasites are degenerating, is associated with an inflammatory reaction of different intensity and is generally when the neurological signs/symptoms present [5]. At radiological level edema and contrast-enhancing ring lesions can be seen. In contrast, in individuals with extraparenchymal lesions, the initial neurological signs/symptoms are more frequently related to the mass effect of the parasites, impacting the normal flux of the cerebrospinal fluid, causing hydrocephalus associated with signs/symptoms of increased intracranial pressure, rather than the inflammation [4,5]. Symptomatic cases of NCC may include acute symptomatic seizures, epilepsy, acute and chronic headaches, and psychiatric disorders such as depression and dementia [7].

Inadequate sanitation and limited awareness of the disease contributes to the perpetuation of the parasite’s life cycle [8]. Other risk factors include low health education and subsequent low health literacy, as well as socioeconomic and environmental conditions, such as poverty, outdoor free-roaming pig rearing, and improper faecal-based land fertilization near homes [9].

NCC is a key contributor to acute symptomatic seizures, epilepsy, acute and chronic headaches and selected psychiatric disorders in African countries, where the parasite is endemic [10]. The global incidence of epilepsy is estimated at 61.4 per 100,000 individuals annually, with higher rates in low-income countries [11]. According to the World Health Organization (WHO), NCC accounts for approximately 30% of epilepsy cases in endemic countries [2,12], impacting up to 1% of the population in Africa, Asia and Latin America [13]. In Latin America, a meta-analysis indicated a significant 15.8% epilepsy incidence [14]. In sub-Saharan Africa, the prevalence of NCC was 22% in people with epilepsy [10].

The diagnosis of NCC involves various degrees of certainty based on different diagnostic tools. The highest degree of diagnostic certainty for NCC can be achieved through the combination of neuroimaging tools, including magnetic resonance imaging (MRI) and computed tomography (CT scan), supported by immunodiagnostic tests for detection of antibodies or antigens and epidemiological data [15]. The enzyme-linked immunoelectrotransfer blot (EITB) assay using lentil lectin purified parasites glycoprotein antigens (LLGP) has sensitivity of 98% and specificity of 100% [16]. However, the commercially available antibody detection assays have lower sensitivity and specificity and frequently cross react with related cestodes such as *Hymenolepis nana* and *Echinococcus* spp. [17]. Of late, the development and validation of a point-of-care test for NCC in rural southern Tanzania and eastern Zambia showed promising results [18]. Unfortunately, in low-income countries such as Mozambique, only a small fraction of the population has access to neuroimaging and appropriate serological testing because of the limited number of available equipment and supplies [19]. NCC is classified based on the updated Del Brutto criteria, which include absolute, neuroimaging, clinical and exposure criteria, allowing two degrees of diagnostic certainty, which is definitive and probable NCC [15].

Treatment of NCC should foremost be based on symptomatic management with anti-seizure medication and corticosteroids to control acute symptomatic seizures/epilepsy and intracranial hypertension, respectively. In most cases of symptomatic parenchymal vesicular NCC, anthelminthic medication, such as praziquantel and/or albendazole in association with corticosteroids, is indicated [20]. When it comes to the colloidal stage, there is controversy on either to treat or let the infections resolves naturally. This is because of the exacerbated inflammatory reactions against degenerating parasites which can result in worsening of neurological signs/symptoms. Other stages of the parasite do not require anthelminthic therapy. Extraparenchymal NCC may need prolonged treatment with anthelminthic medication and corticosteroids [20]. In few cases surgery may be required to control hydrocephalus or excise large cysts or cyst masses [7,21,22]. There is a recent report that a combination of praziquantel and albendazole together with corticosteroids for symptomatic parenchymal vesicular NCC in an African population may be more effective than monotherapy, i.e., praziquantel and albendazole alone, although so far treated cases are few and potential side-effects have not been investigated over the longer term in sufficient numbers, hence caution, especially in resource-limited settings, needs to be exerted [23].

CC and NCC are substantial public health and agricultural concerns in Mozambique, particularly prevalent in rural regions [24]. Risk maps for CC and abattoir records, indicate that the central, north and northeast areas of the country might carry a higher risk for the occurrence of porcine and human CC than other areas [25]. Nevertheless, NCC is still an unknown entity for most healthcare providers, an underdiagnosed and neglected disease [24,26–28]. Some studies in Mozambique have demonstrated an antibody seroprevalence of *Cysticercus cellulosae* ranging from 14.9-20.8% in populations of apparently healthy children and adults [25,29,30]. In people with psychiatric disorders, the seroprevalence to *C. cellulosae* antibodies was even higher, ranging from 7.6-51.4% [25,28]. These data suggest that CC and NCC may be highly prevalent in some areas of Mozambique and information provided in previous paragraphs outlines that cases of epileptic seizures/epilepsy as well as other neurological disorders such as acute and chronic headaches may be related to NCC in *T. solium* endemic areas.

Thus, our study aimed to determine the prevalence of CC and NCC as well as that of seizure activity and chronic headaches in the Mocuba district, Zambézia province, central Mozambique, and to explore their associations.

## Methods

### Ethics statement

The study was approved by the Mozambique National Bioethical Committee of Health (51/CNBS/2017) and followed the principles of the Helsinki Declaration. It also received endorsement from administrative authorities in Zambézia province and local community leaders. Additionally, the research obtained approval from the ethics committee of the TUM University Hospital, Technical University of Munich (TUM), Germany, under the reference number 537/18 S-KK.

Prior to the initiation of the study, participants over 18 years of age were asked to provide a written informed consent. Written informed consent was also obtained from the parent or legal guardian of each participant below 18 years of age. Besides parental consent, participants aged 12–17 years provided their assent. Those participants that were illiterate added their fingerprint to the consent form along with an impartial witness who also signed and was present during the informed consent process. We kept all information of the participants strictly confidential.

### Study area and population

**Fig 1:**
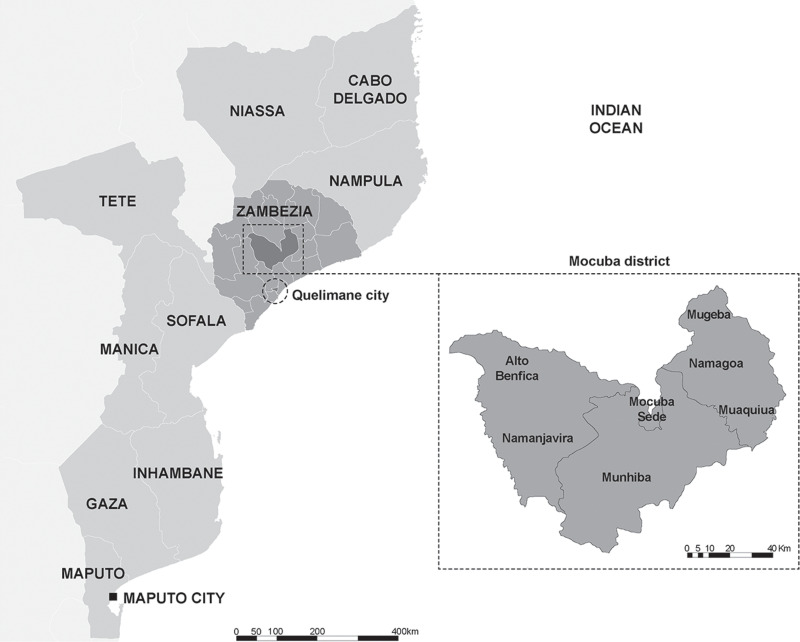
Geographic representation of the study site in Mozambique *(Created by Patricia Noormahomed with Adobe Illustrator, based on LandsatLook Viewer Map (* https://landsatlook.usgs.gov).

The community-based study was conducted within six villages situated in the Mocuba district of Zambézia province, located in central Mozambique and the clinic-based study was conducted in the Central Hospital of Quelimane city in Quelimane district. Fig 1 shows the geographical location of the communities included in the study.

The Mocuba district is notable for its dense pig population, commonly raised in suboptimal sanitary conditions [24]. Furthermore, Zambézia province faces the challenge of preventable infectious diseases, including several neglected tropical diseases (NTDs), such as lymphatic filariasis, onchocerciasis, schistosomiasis, soil transmitted parasites, malaria, and HIV/AIDS [31–36].

### Study design and sampling

**Fig 2:**
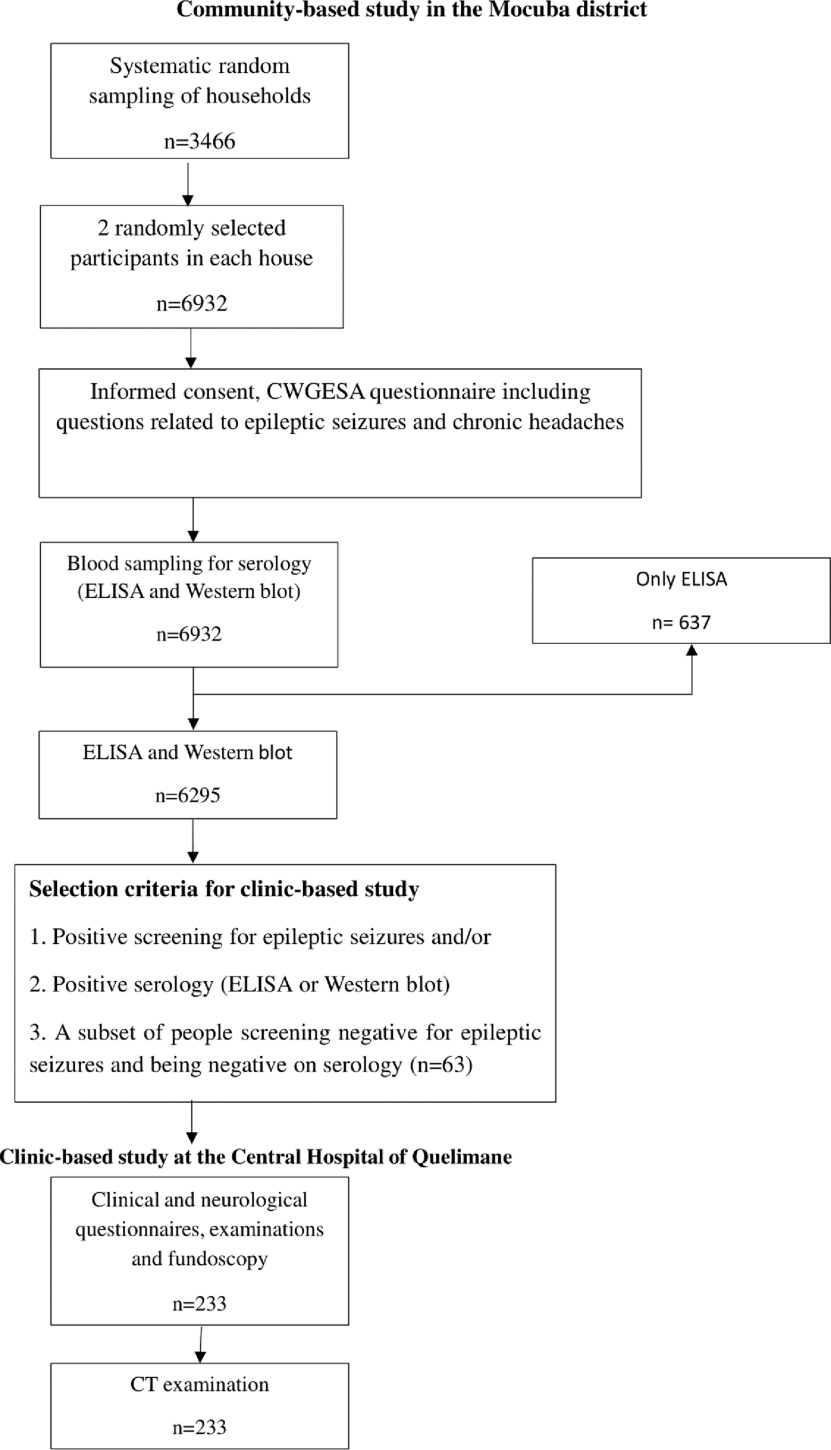
Study sampling flowchart. ELISA = enzyme-linked immunosorbent assay for the detection of cysticercus antigen; CT = computed tomography; NCC = neurocysticercosis.

Our study adopted a cross-sectional design that combined a community-based (6,932 participants) from six villages and a clinic-based study including 233 individuals selected as described in Fig 2. It was conducted among residents of Mocuba district from July 2018 to June 2019.

#### Community-based study.

The community-based study phase utilized the same methodology that was described by Langa et al. [24]. Basically, the sampling frame was constructed using information of the "Mozambique National Institute of Statistics" [31] and based on the assumption that 15% to 18.6% (p1, p2) of households would have at least one member with cysticercosis according to a study done in northwestern district of Mozambique, Angónia [28]. Thus, assuming a 95% confidence level, power of 80% (β), we calculated the study sample size using the formula $n^{\prime }={\frac{\left[z_{1-\alpha /2}{\left\{\left(r+1\right)\overset{―}{pq}\right\}}^{1/2}+z_{1-\beta }{\left(rp_{1}q_{1}+p_{2}q_{2}\right)}^{1/2}\right]}{r{\left(p_{1}-p_{2}\right)}^{2}}}^{2}$ [37].

The sampling procedure followed a household-based design, in two stages, where Primary Sampling Units (PSUs) consisted of 6 villages, and households were chosen during the second stage totaling 3,466 households. The selection utilized a probability proportional to size (PPS) approach and two participants were chosen from each household, resulting in a total of 6,932 participants. This approach aimed to maintain equal participation probability across the study.

The study team consisted of research professionals, including doctors (EVN; LG), nurses, and interviewers, who explained the study’s objectives to participants and obtained written informed consent. Those who were unable to sign their consents provided their fingerprints. After informed consent was obtained, individuals were presented with a comprehensive questionnaire developed by the Cysticercosis Working Group for Eastern and Southern Africa (CWGESA) with modifications as described in the previous study by our group [24]. The questionnaire covered questions referring to history of seizure activity and chronic headaches but also to sociodemographic factors and investigated participant’s awareness of habits and practices related to the transmission risk of *T. solium*. Furthermore, for the clinical aspect of the study we collected clinical and neurological data through a specific questionnaire. Additionally, each participant was invited to give blood for serological diagnosis of CC as described below.

As there was no neurologist on site and it was therefore difficult to distinguish seizures such as acute symptomatic seizures from epilepsy, in addition to the stigma attached to a diagnosis of epilepsy, we opted for the more comprehensive term of seizures as opposed to non-seizures such as pseudo-seizures. Individuals who answered positively to any of the screening questions referring to seizures in the CWGESA questionnaire were given a diagnosis of seizures. However, this included seizures in the broader sense, i.e., generalized and focal seizures. To assess chronic headaches, participants were asked if they had a history of daily headache that occurred for at least 15 consecutive days and for more than three months. The formal diagnoses of seizures, including whether generalized or focal, and chronic headaches were reached jointly by the study team through consensual discussions based on previously developed clinical case vignettes [24] and were confirmed by the psychiatric technicians (3) assigned to the study. Patients who screened positive for seizures and chronic headaches in the CWGESA questionnaire were referred to the health center of the study villages for diagnosis confirmation, treatment and follow-up. Of note, although we included generalized and focal seizures in the community-based study (n = 928), which were classified as ‘screening positive’, we took a more conservative approach to calculating prevalence estimates by including only generalized seizures (n = 448).

**Fig 3:**
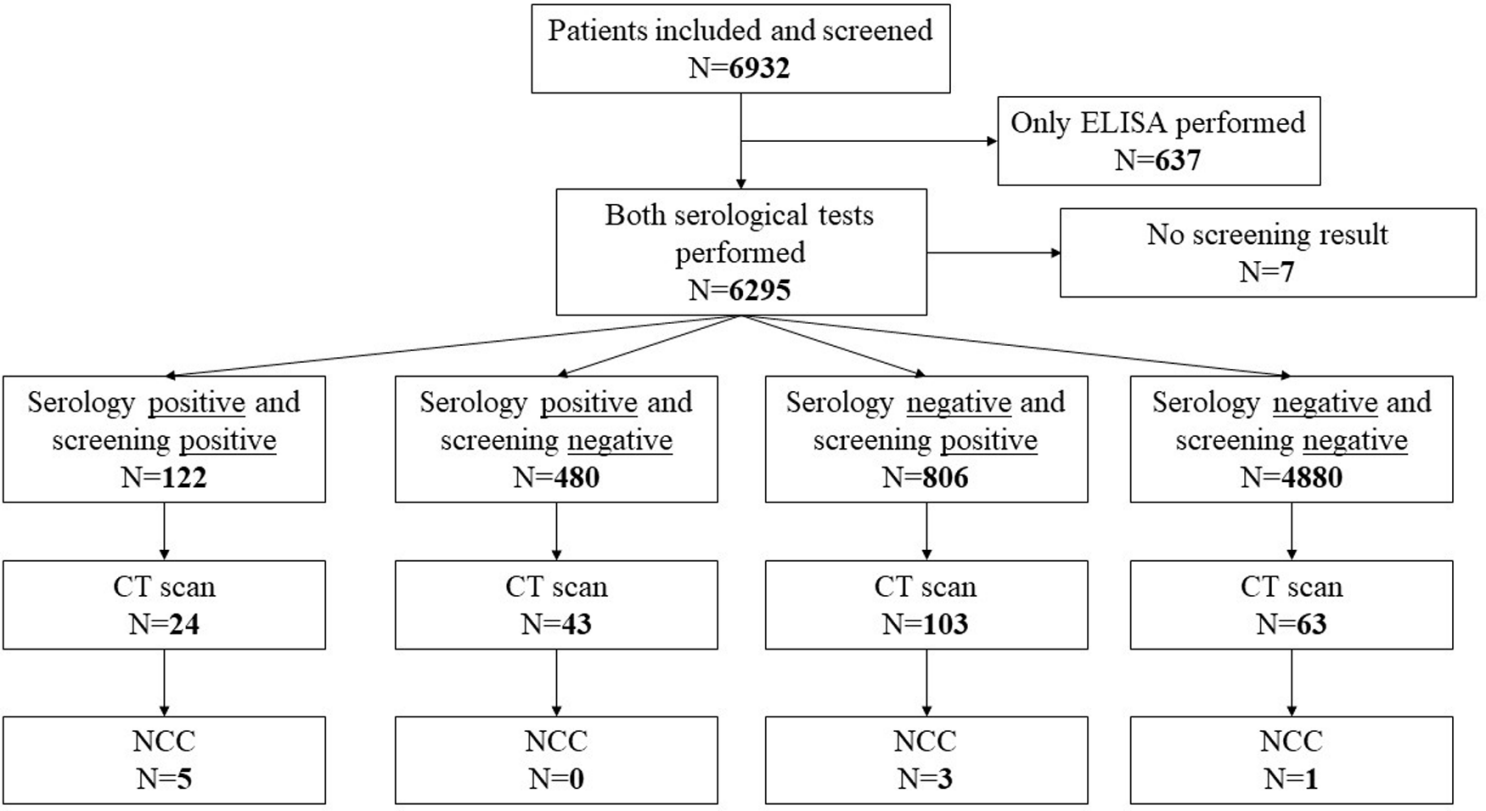
Categorization of patients into different groups based on cysticercosis serology and screening epilepsy. ELISA = enzyme-linked immunosorbent assay for the detection of cysticercus antigen; CT = computed tomography; NCC = neurocysticercosis.

For a visual representation of the study sampling process, please refer to Figs 2 and 3.

#### Blood sampling and processing.

A 5 ml venous blood sample was drawn from each participant via venipuncture after consenting, placed in a dry tube and transported immediately in cooling boxes at 4°C to the Mocuba District Hospital Laboratory where they were centrifuged for 5 min at 1500 r.p.m. to obtain serum. Serum was stored at -20°C until shipment to the Parasitology Laboratory at Faculty of Medicine, Eduardo Mondlane University in Maputo, where samples were stored at -80°C until further processing following the protocol detailed in our previous study [24].

#### Serological testing.

*T. solium* larval (cysticercus) antigen (Ag) and antibodies (Abs) were assessed using commercial Enzyme-Linked Immunosorbent Assay (ELISA) Kit (ApDia, Belgium) for Ag and Western blot Kit (LDBIO, Lyon, France) for Abs at the Parasitology Laboratory, Faculty of Medicine, Eduardo Mondlane University in Maputo, according to manufacturer’s instructions, consistent with our previous study [24].

The monoclonal antibody-based B158C11A10 ELISA (Cysticercosis Ag-ELISA, ApDia, Belgium) was utilized to detect circulating *T. solium* Ag. The Ag-index was calculated by dividing the mean optical density (OD) of each serum sample by the cut-off value. Detection of IgG Abs against *T. solium* larva was done through Western Blot (LDBIO Diagnostic, Lyon, France), with positivity determined by the presence of at least 2 bands (6–8 kDa; 12 kDa; 23–26 kDa; 39 kDa; 50–55 kDa), following the manufacturer’s instructions.

#### Clinic-based study at the Central Hospital of Quelimane.

We recruited 233 participants for clinical/neurological and cerebral CT examination from the community-based study. The recruitment was done by convenience according to participants’ availability and according to the four groups detailed in Fig 3. The number of 233 was determined by financial restrictions mainly due to CT examinations. Participants diagnosed in the community-based study as people with seizures and/or were positive on serological results for *T. solium* larvae Abs or Ag were included after obtaining consent. Additionally, a random subset of 63 participants who were serologically negative and did not report seizures or chronic headaches were sent for CT examination (Fig 3). These participants were transported to the Central Hospital of Quelimane (HCQ), 155 km from Mocuba District Hospital. At HCQ, the participants were subject to clinical and neurological examination including eye fundoscopy to confirm the CWGESA questionnaire-based diagnosis of seizures and chronic headaches and to exclude possible co-morbidities. This was performed by a multidisciplinary team, including experts in internal medicine, pediatrics, psychiatry, ophthalmology, and radiology, Additionally, participants underwent cerebral CT examination to detect NCC-related brain lesions. Patients with co-morbidities (malaria, HIV/AIDS) were treated as per Mozambique Ministry of Health guidelines [38]. All people with seizures had their diagnosis confirmed and in some cases their medication was adjusted for subsequent follow-up in their health units. Standard anti-seizure medication used included Phenobarbital, Phenytoin and Carbamazepine.

The cerebral CT examination was conducted using Siemens equipment, model NR 86693, SN-10165880 (Siemens Medical Solutions, Erlangen, Germany), manufactured in 2014, featuring 16mm thick slices. At the time the study was conducted radiographic contrast dye was not available. Female participants of reproductive age underwent a urine pregnancy test prior to the CT examination. For the diagnosis of NCC on CT scans we used the following criteria: patients with at least one viable or degenerating lesion were classified as having active NCC; patients with only calcified NCC-typical lesions were classified as having calcified NCC [39,40]. The CT scans were sent to the Department of Neuroradiology at the Technical University of Munich, where they were read by experts.

### Statistical analysis

Data were entered into EpiData Entry Client version 4.3 and analyzed using STATA version 13.0 (Stata Corp., College Station, TX) and R version 4.4.1. Initial analysis encompassed descriptive measures such as calculating frequencies and percentages for categorical variables. Age was stratified into distinct groups (2–14, 15–24, 25–54 and ≥55 years). Logistic regression models were employed to evaluate the association between seizures and chronic headaches, on the one side, and seropositivity for *T. solium* cysticercosis, on the other side, both in unadjusted and adjusted formats accounting for age, sex, education and occupation. NCC prevalence proportions were assessed for the entire population and for the subset of individuals screening positive for seizures. Results derived from serology were extrapolated to account for differences in selection for CT examination. We report the number and the prevalence of NCC extrapolated to the entire study population under the assumption that every participant had a CT examination. A significance level of 5% (alpha error) was adopted.

The reporting of the study followed the STROBE checklist, S1 File.

## Results

### Sociodemographic characteristics and risk factors for cysticercosis

The age of the 6,932 participants included in the study ranged from 2 to 87 years, (S1 Fig) with a mean age of 27 years (SD ± 18 years). Table 1 provides an overview of the sociodemographic characteristics of the participants. The majority of participants, (56.7%, n = 3,896) were female and reported no prior knowledge of taeniasis (93.8%, n = 6,505). Half of the participants completed primary school and relied on agriculture as their primary source of income. Most of the participants (59.6%) consumed fountain water, while waste treatment was predominantly carried out through the use of latrines, accounting for 81.5% of the participants. A total of 17.9% (1,241) of the participants reported that they practiced open defecation. Regarding dietary habits, a significant proportion of the participants, 72.6%, consumed pork meat, with the majority, 71.6%, acquiring it from in informal markets. Additionally, 24.3% of households reported pigs roaming in their vicinity, primarily in the village around their own homes.

**Table 1:** Sociodemographic characteristics of the study participants.

Variables	Category	Overall population Frequency (%)	Clinical study population Frequency (%)
N		6932	233
Village	Alto-Benfica	629 (9.1)	26 (11.2)
	Mocuba - Sede/Municipality	2393 (34.5)	59 (25.3)
	Muaquiua	538 (7.8)	24 (10.3)
	Mugeba	2363 (34.0)	90 (38.7)
	Munhiba	627 (9)	15 (6.4)
	Namanjavira	382 (5.5)	19 (8.2)
Sex	Male	3032 (43.7)	105 (45.1)
	Female	3896 (56.2)	128 (54.9)
Age group	<15 years	2035 (29.4)	64 (27.5)
	15-24 years	1586 (22.9)	43 (18.5)
	25-54 years	2646 (38.2)	97 (41.6)
	≥55 years	665 (9.6)	29 (12.4)
Education	No formal education	1010 (14.6)	41 (17.6)
	Primary education	4976 (71.8)	166 (71.2)
	Secondary or higher education	945 (13.6)	26 (11.2)
Occupation	Market trader	165 (2.4)	1 (0.4)
	Farmer	3680 (53.1)	129 (55.4)
	Student	2082 (30)	59 (25.3)
	Public service	124 (1.8)	5 (2.1)
	Other	880 (12.7)	39 (16.7)
Water source	River	814 (11.7)	44 (18.9)
	Fountain	4132 (59.6)	118 (50.6)
	Bore hole	1824 (26.3)	66 (28.3)
	Cistern	153 (2.2)	1 (0.4)
	Other	8 (0.1)	4 (1.7)
Waste disposal practices	Latrine	5650 (81.5)	199 (85.4)
	Open defecation	1241 (17.9)	33 (14.2)
	Toilet	37 (0.5)	1 (0.4)
Pork consumption	Yes	5032 (72.6)	172 (73.8)
	Cooked	5016/5032 (99.7)	171/172 (99.4)
	Fried	9/5032 (0.2)	1/172 (0.6)
	Baked	7/5032 (0.1)	0/172 (0)
	No	1900 (27.4)	35 (17.9)
Sources of pork purchase	Informal market	4962/5029 (99.6)	168 (97.7)
	Butcher	17/5029 (0.3)	1 (0.6)
	Other	52/5029 (1)	3 (1.7)
Pig farmer	Yes	670 (9.7)	31 (13.3)
	Keeping pigs in pig pens	173/606 (31.4)	5/31 (16.1)
	Letting pigs roam freely	433/606 (68.6)	26/31 (83.9)
	No	6260 (90.3)	202 (86.7)
Knowledge of taeniosis	Yes	427 (6.2)	17 (7.3)
	No	6505 (93.8)	216 (92.7)
Ever had nodules under the skin	Yes, now	47 (0.7)	1 (0.4)
	Yes, within last year	66 (1)	4 (1.7)
	Yes, more than a year ago	111 (1.6)	9 (3.9)
	No	5997 (86.5)	197 (84.9)
	Does not know	709 (10.2)	21 (9.1)
Sanitation facilities	No latrine	1218 (17.6)	29 (12.4)
	Latrine exists and is completely protected	2950 (42.6)	100 (42.9)
	Latrine exists and is partially protected	1941 (28)	66 (28.3)
	Latrine exists but is not protected	823 (11.9)	38 (16.3)
Recent utilization of available latrine	Yes	4338/5714 (75.9)	146/209 (69.9)
	No	1376/5714 (24.1)	63/209 (30.1)

### Community-based study

#### Association between seizures, cysticercosis and sociodemographic factors.

The results of seizures and CC serology and their association with sociodemographic characteristics are summarized in Table 2. In general, out of the 6,932 participants included, 6.5% (448/6,932) screened positive for seizures and 9.6% (606/6,295) had positive serology for at least one anti-cysticerci test (Ag-ELISA or Western blot).

**Table 2:** Prevalence of seizures and seropositivity to cysticercosis with reference to sociodemographic factors.

Variables	Category	Screening positive for seizures^£^ n/N (%)	Serology positive^†^ n/N (%)
N		448/6932 (6.5)	606/6295 (9.6)
Village	Alto-Benfica	20/629 (3.2)	17/124 (13.7)
	Mocuba municipio	148/2393 (6.2)	158/2378 (6.6)
	Muaquiua	29/538 (5.4)	29/455 (6.4)
	Mugeba	89/2363 (3.8)	307/2346 (13.1)
	Munhiba	44/627 (7)	33/607 (5.4)
	Namanjavira	18/382 (4.7)	62/382 (16.2)
Sex	Male	204/3032 (6.7)	298/2745 (10.9)
	Female	244/3896 (6.3)	308/3543 (8.7)
Age group	<15 years	185/2035 (9.1)	101/1844 (5.5)
	15-24 years	89/1586 (5.6)	124/1432 (8.7)
	25-54 years	141/2646 (5.3)	293/2398 (12.2)
	≥55 years	33/665 (5)	88/618 (14.2)
Education	No formal education	101/1010 (10)	86/921 (9.3)
	Primary education	313/4976 (6.3)	453/4453 (10.2)
	Secondary or higher education	34/945 (3.6)	67/917 (7.3)
Occupation	Farmer	185/3680 (5)	415/3306 (12.6)
	Other	263/3251 (8.1)	191/2985 (6.4)
Water source	River	83/814 (10.2)	79/728 (10.9)
	Fountain	238/4132 (5.8)	368/3706 (9.9)
	Bore hole	119/1824 (6.5)	150/1700 (8.8)
	Cistern	7/153 (4.6)	7/149 (4.7)
	Other	1/8 (12.5)	2/8 (25)
Waste disposal practices	Latrine	368/5650 (6.5)	470/5182 (9.1)
	Open defecation	76/1241 (6.1)	133/1069 (12.4)
	Toilet	3/37 (8.1)	3/37 (8.1)
Sanitation facilities	No latrine	80/1218 (6.6)	121/1048 (11.5)
	Latrine exists and is completely protected	170/2950 (5.8)	218/2700 (8.1)
	Latrine exists and is partially protected	140/1941 (7.2)	167/1779 (9.4)
	Latrine exists but is not protected	58/823 (7)	100/765 (13.1)
Recent utilization of latrine	Yes	254/4338 (5.9)	358/3926 (9.1)
	No	136/1814 (7.5)	158/1667 (9.5)
Pork consumption	Cooked	342/5017 (6.8)	481/4624 (10.4)
	Fried	1/9 (11.1)	1/9 (11.1)
	Baked	0/7 (0)	0/7 (0)
	No	105/1900 (5.5)	124/1653 (7.5)
Sources of pork purchase	Informal market	338/4961 (6.8)	473/4570 (10.4)
	Butcher	1/17 (5.9)	0/16 (0)
	Other	4/52 (7.7)	9/52 (17.3)
Pig farmer	Yes	72/670 (10.7)	69/600 (11.5)
	No	375/6259 (6)	537/5690 (9.4)
Knowledge of taeniosis	Yes	27/427 (6.3)	55/407 (13.5)
	No	421/6505 (6.5)	551/5885 (9.4)
Ever had nodules under the skin	Yes, now	5/47 (10.6)	6/46 (13)
	Yes, within last year	5/66 (7.6)	7/61 (11.5)
	Yes, more than a year ago	8/111 (7.2)	14/94 (14.9)
	No	382/5997 (6.4)	524/5435 (9.6)
	Does not know	48/709 (6.8)	55/654 (8.4)
Pig habitat	Pig pens	12/124 (9.7)	16/112 (14.3)
	Roam freely	39/507 (7.7)	34/470 (7.2)

£ Refers to at least one positive answer to the seizure screening questions in the CWGESA questionnaire.

^†^Seropositivity was defined as ELISA and/or immunoblot positivity. ELISA and Immunoblot results together were available for only 6295 participants; for the remaining participants, only ELISA results were available.

The prevalence of seizures ranged from 3.2% (20/629) for Alto Benfica village to 7% (44/627) in Munhiba. On the other hand, the CC seroprevalence varied between villages from 5.4% (33/607) in Munhiba to 16.2% (62/382) in Namanjavira.

CC seropositivity was higher in males (10.9%) which increased with age. Specifically, CC seropositivity was lower in children under the age of 15 years (5.5%) and higher in adults above the age of 55 years (14.2%). Among different occupations, farmers had the highest CC seropositivity at 12.6% (415/3,306).

Furthermore, the assessment of environmental sanitation, revealed that individuals who sourced their water from the river had a higher CC seroprevalence at 10.9% (79/728) as well as prevalence of seizures at 10.2% (83/814). Additionally, CC seropositivity was higher among those who engaged in outdoor defecation, with a rate of 12.4% (133/1069), who had no latrines or unprotected latrines, and among those who consumed pork and purchased it from informal markets at 10.4% (482/4639).

#### Association between seizures, chronic headaches and cysticercosis seroprevalence.

In the univariable logistic regression, we observed that seizures and chronic headaches were associated with 51% and 34% higher odds, respectively, of testing serologically positive for CC (seizures cOR: 1.51, p < 0.001, 95%CI: 1.12–2.00 and chronic headaches cOR: 1.34, p < 0.001, 95%CI: 1.13–1.59; Table 3). Similar findings were observed in the adjusted logistic regression model (seizures (aOR=1.64, 95%CI 1.21–2.19) and chronic headaches (aOR=1.21, 95%CI 1.01–1.44)).

**Table 3:** Associations between seizures, chronic headaches and cysticercosis seroprevalence.

Symptom	Category^£^	Serology			Odds Ratio (95%CI)	
		Overall	Negative	Positive	unadjusted	adjusted
n		6295	5693 (90.4)	602 (9.6)		
Chronic headaches	No	3259	2986 (91.6)	273 (8.4)	Reference	Reference
	Yes	2881	2566 (89.1)	315 (10.9)	1.34 (1.13–1.59)	1.21 (1.01–1.44)
	Don’t know/NA	155	141 (91.0)	14 (9.0)		
Seizures	No	5855	5311 (90.5)	544 (9.5)	Reference	Reference
	Yes	433	375 (86.6)	58 (13.4)	1.51 (1.12–2.00)	1.64 (1.21–2.19)
	Don’t know/NA	7	7 (100.0)	0 (0.0)		

95%CI = 95% confidence intervals; £ Don’t know/NA were excluded from the analyses of the Odds Ratios.

### Clinic-based study at the Central Hospital of Quelimane

Of the 6,932 participants in the community-based study, a subset of 233 participants was selected and referred to HCQ for their clinical/neurological and CT examinations. The sociodemographic characteristics of the study participants are summarized in Table 1. There were no marked differences compared with the overall study population in terms of demographic factors and factors associated with *T. solium* infection.

#### Results of computed tomography examinations.

We categorized participants into four groups, as illustrated in Fig 3. Out of 233 participants who underwent CT examination, 9 (3.9%) exhibited brain lesions consistent with NCC – 5 of the 24 people who screened positive for seizures and were positive in at least one CC serological test; 3 of the 103 people with negative CC serology but who screened positive for seizures and 1 of 63 patients who both screened negative for seizures and had negative CC serological test results. Notably, one of the patients with active NCC was a child under the age of 14.

Screening positive or negative refers to the screening questions for seizures within the CWGESA questionnaire and hence the broader screening for seizures (focal and generalized seizures; see methodology).

Among the 9 patients with NCC, 2 had active NCC lesions (both screened positive for seizures and had positive CC serology in ELISA and Western blot), while 7 had multiple calcified lesions (6 screened positive for seizures, 4 were serologically negative in both tests, one positive in both tests, one positive in immunoblot and one positive in ELISA, Table 4).

**Table 4:** Seropositivity for cysticercosis and results of computed tomography examinations by subgroup.

	Population	CT performed	NCC
Total	6288	233	9 (3.9%)
**Serology positive and screening positive for seizures**	**122**	**24**	**5 (20.8%)**
* ELISA and immunoblot positive*	*16*	*5*	*3 (60%)*
* ELISA positive only*	*58*	*14*	*1 (7.1%)*
* Immunoblot positive only*	*48*	*5*	*1 (20%)*
**Serology positive only**	**480**	**43**	**0 (0%)**
* ELISA and immunoblot positive*	*42*	*8*	*0 (0%)*
* ELISA positive only*	*226*	*33*	*0 (0%)*
* Immunoblot positive only*	*213*	*2*	*0 (0%)*
**Screening positive for seizures only**	**806**	**103**	**3 (3.0%)**
**Serology negative and screening negative for seizures**	**4880**	**63**	**1 (1.6%)**

#### Serological comparisons between people with seizures and those without seizures.

Serological findings differed significantly between individuals with seizures and those without. Among participants with seizures, 58/433 (13.4%) showed at least one positive CC serological result, whereas in those without seizures, this was 544/5855 (9.3%; (p < 0.001). The majority of the total sample (5686/6288, 90.4%) tested negative for both CC serological tests. Moreover, 27 (6.2%) of individuals with seizures exhibited positivity to Ag-ELISA, a higher proportion than the 256 (4.4%) observed in people screening negative for seizures, S1 Table.

#### Neurocysticercosis prevalence.

In the clinic-based study population of 233 participants, 9 (3.9%) individuals had NCC of any type and 2 (0.9%) participants had active NCC. Accounting for the study design the NCC prevalence yielded 2.0% among the entire study population (0.4% active NCC; Among the 433 people who screened positive for seizures, 6.1% were estimated to have NCC (1.6% active NCC), S2 Table.

## Discussion

Our large community-based study of 6,932 participants with sociodemographic, clinical/neurological and a subset of these with neuroradiological data provides robust community-based prevalence estimates of seizures, chronic headaches, CC and NCC in Mocuba district, Zambézia province, although the limitations with data referring to chronic headaches are acknowledged. The positive and significant association between seizures and chronic headaches, on the one side, and positive CC serology, on the other side, confirms that conditions are interlinked. In addition, the study findings corroborate the frequent occurrence of CC in Mozambique in all age groups, including children, as has been shown in other African countries [19,24,41,42] and re-affirm common risk factors for CC, such as being male, being older, being a farmer, practicing open defecation, consuming pork and purchasing it from informal markets [9].

### Community-based study

The observed community-based prevalence estimates of seizures and CC of 6.5% and 9.6%, respectively, in Mocuba district, Zambézia province, were high. For CC, a recent review by Zulu et al. 2023 allows to put our data on CC seroprevalence into perspective within the African context, indicating that our prevalence estimates lie within the middle range [43]. The prevalence estimates of seizures was very high, although a conservative approach was chosen and only generalized seizures were included. In a recent representative review and meta-analysis, the overall lifetime prevalence of epilepsy was 7.60 per 1,000 population (95% CI 6.17–9.38) and was higher in low-income and middle income countries (8.75 per 1,000; 95% CI 7.23– 10.59) than in high-income countries (5.18 per 1,000; 95% CI 3.75–7.15) [5]. In our study, we could not confirm whether the seizures we diagnosed with the CWGESA questionnaire justified the diagnosis of epilepsy, which could be the reason for the high prevalence in our study.

There was also a positive association of CC serology and both seizures and chronic headaches. Whereas the association of CC and seizures/epilepsy is well known, there is only little evidence about the association of CC and chronic headaches. These findings are reinforced by both crude and adjusted logistic regression analyses, underscoring the robustness of the observed associations. Although the community-based prevalence of chronic headaches with 46.2% seems to be overestimated compared to other community-based prevalence estimates for chronic headaches, e.g., the prevalence of tension-type headache in communities of northern Tanzania which was 7% during the previous year [44], a previous study from our group found a statistically significant association between Ag-ELISA seropositivity for *T. solium* and chronic headaches using the same screening questions than in our study [24]. This represents an interesting finding as usually the emphasis of neurological disorders in association with CC/NCC is on seizures/epilepsy and not on headaches.

The variation in prevalence estimates of seizures (3.2%-7%) and CC (5.4%-16.2%) across different villages suggests that local practices and other risk factors may play a role in disease occurrence. These include differences in healthcare access, lack of awareness of *T. solium* taeniasis/CC/NCC and its transmission, genetic predisposition, socioeconomic and environmental conditions such as poor treatment of drinking water, deficient waste disposal, open defecation, use of latrines without protection, or specific cultural practices related to health and well-being, as well as co-morbidities that warrant further investigation [24]. The presence of some of those risk factors was previously observed in Angónia district of Mozambique [26,28] and other regions of Africa [45]. The higher seropositivity in males may indicate different exposure patterns or susceptibility to CC/NCC. Moreover, the higher seropositivity among adults above 55 years might reflect long-term exposure to risk factors, cumulative infections, or alterations in immunity over time. These factors were previously described in other studies as risk factors that increase the prevalence in low-inccome and middle-income countries [1,46]. Our study findings also indicate that farmers, due to occupational exposure, may have a higher seropositivity rate, emphasizing the need for occupational health measures. Agricultural practices, contact with animals and environmental contamination may contribute to higher infection rates in this group. Additionally, the relationship between environmental sanitation and seropositivity highlights the role of waterborne transmission and poor sanitation practices. Furthermore, higher seropositivity among those consuming pork from informal markets aligns with the known transmission pathways of CC/NCC and underscores the need for food safety measures to reduce the risk of infection through contaminated meat consumption [47]. The awareness and knowledge of a multitude of risk factors for the transmission of *T. solium* in relation to human, animal and environmental health, as well as the health of the ecosystem as a whole, once again emphasizes the need for One Health approach [48].

### Clinic-based study

In our community-recruited, clinic-based study population of 233 individuals, 3.9% of participants had NCC-typical cerebral lesions on cerebral CT scan, most of them in the calcified stage, which extrapolated to the entire study population and that of seizures only results in an NCC prevalence of 2% and 6%, which is rather low for a *T. solium* endemic region. In fact, the NCC prevalence in our study population was less than 10% of that in a similar study in Angónia district of Mozambique, which was a staggering 15.5% [28]. The huge difference between the two studies of adjacent regions of Mozambique may be explained by the local difference in freely roaming pig populations, by the study design and by the time the study was conducted, which was 10 years earlier than our study. It is also lower than reported in a systematic review and meta-analysis including studies from different geographies estimating NCC to be responsible for more 22% of people with epilepsy in *T. solium* in Sub-Saharan Africa [10]. Most of our patients with NCC had seizures (8/9) and had lesions in the calcified stage only (7/8). It is known that the presence of parenchymal calcifications may be linked to the occurrence of acute symptomatic seizures due to inflammatory reactions manifested by perilesional edema or to epilepsy based on the epileptogenicity of calcifications [49,50]. This finding aligns with similar observations reported in studies conducted across various regions globally [51].

Interestingly, 4 of the 9 participants with confirmed NCC had negative CC serology and all had lesions in the calcified stage only. A lack of sensitivity of anti-cysticerci serological tests, both for Ag and Abs, especially in the context of calcified lesions has been demonstrated previously and can make community-based studies challenging, especially the selection of individuals who need to undergo neuroimaging [52]. However, generally more people tested positive in people with seizures, particularly for Ag-ELISA, which may suggest that some of these cases had NCC.

The presence of active NCC lesions in a child under the age of 14 is of particular concern, as it highlights the vulnerability of younger populations to NCC. Before this study, we reported a series of 3 patients, aged 7–11 years, with a history of epilepsy and/or recurrent headache referred from the same district to the Central Hospital of Quelimane that were confirmed to be associated with NCC after cerebral CT examination [19]. This emphasizes the need for targeted messages in awareness campaigns and education to promote prevention and early detection in children and adolescents.

Overall, the findings of our clinic-based study have important implications for clinical management and public health approaches in rural settings such as Mocuba district. Enhanced awareness campaigns, targeted educational programs, and early diagnosis and treatment initiatives can play a crucial role in reducing the burden of seizures/epilepsy and NCC in the region.

### Study limitations

While our study contributes crucial insights, certain limitations warrant acknowledgment. The cross-sectional design limits our ability to establish a causal relationship rather than an association and the regional scope of our study necessitates cautious extrapolation to broader populations. In addition, the recruitment of our participants into the clinic-based part of our study was done by convenience according to participants’ availability and according to the four groups detailed in Fig 3. Most studies of NCC strictly recruit people with epilepsy and therefore their reported NCC prevalence may be higher. Also, the prevalence of seizures and in particular that of chronic headaches may have been overestimated due to the rather non-discriminatory screening questions and the unlimited period of time the questions referred to. Usually, people who screen positively on a screening questions will be seen by a specialist neurologist who confirms or rejects the diagnosis, this normally brings down the prevalence estimates.

Furthermore, the choice of imaging technique impacts the sensitivity of lesion detection. While non-contrast-enhanced cerebral CT is useful for identifying vesicular and calcified cysts, it may overlook small cysts with and without edema, and is not very sensitive to extraparenchymal NCC, leading to false negative results. On the other hand, magnetic resonance imaging (MRI) is a powerful tool for diagnosing certain types of lesions such parenchymal cysts of different sizes but also extraparenchymal NCC, such as subarachnoid and intraventricular pathology, however, it may be less sensitive in detecting small calcifications [52]. MRI was not available to our study population. Therefore, the prevalence of NCC reported here, and in particular that of active NCC, may be underestimated.

Similarly, the serological assays used have their limitations in terms of sensitivity and specificity as well as on the discrimination of past or current infection [53]. We also did not perform serological screening for other relevant pathogens such as malaria, toxoplasmosis, toxocariasis and onchocerciasis known to be endemic in the country and the region [24,27,28,32,41,54,55] and that may cause neurological signs/symptoms.

## Conclusions

Our large-scale study provides significant insights into the community-based prevalence of seizures, chronic headaches CC and NCC and their mutual associations, as well as relevant risk factors throughout different communities in Mocuba district, Zambézia province, central Mozambique. Our findings call for targeted interventions and public health measures in specific communities and unscore the significance of accurate diagnostic tools in identifying CC (serological tests) and NCC (neuroimaging). Future research should focus on implementing comprehensive disease surveillance in a One Health approach to access the real burden of CC and NCC and its role in the development of acute symptomatic seizures and epilepsy as well as acute and chronic headaches. We highly recommend to also improve health literacy for *T. solium* taeniasis/CC/NCC among healthcare workers as well as the general population to improve case management and prevention of pathogen transmission to humans, animals and the environment.

## Supporting information

S1 File STROBE Statement—Checklist of items that should be included in reports of cross-sectional studies. (DOCX)

S1 Fig Presents histogram of ages of the study population. (DOCX)

S1 Table Comparison of serological results and epilepsy screening between different groups. (DOCX)

S2 Table Neurocysticercosis prevalence: observed, extrapolated, and post-stratified estimates. (DOCX)
