# Supplementary figures and images for: Cysticercosis and neurocysticercosis in people from Mocuba district, Zambézia province: A Mozambican community-based study
Source: PLoS Negl Trop Dis. 2025 May 13;19(5):e0013083. doi: 10.1371/journal.pntd.0013083 (PMC12119002; doi:10.1371/journal.pntd.0013083)

**S1 Fig.** Presents histogram of ages of the study population.


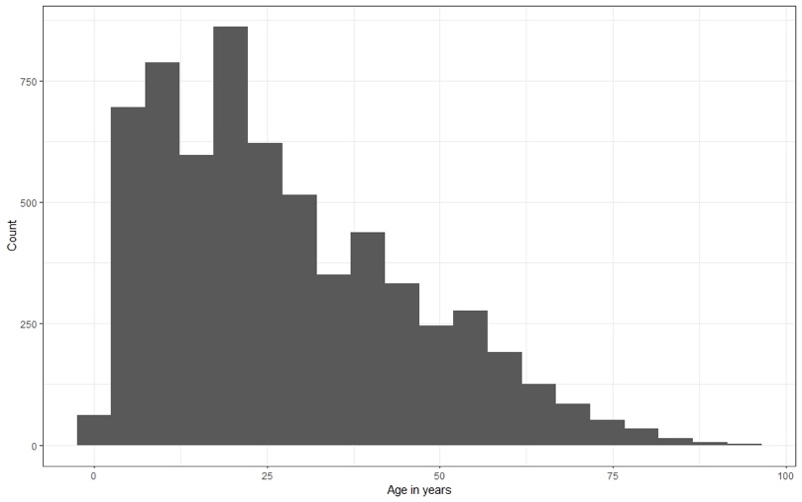

Supplement: S1 Fig — (DOCX) [file pntd.0013083.s002.docx]
