# Supplementary material for: Cysticercosis and neurocysticercosis in people from Mocuba district, Zambézia province: A Mozambican community-based study
Source: PLoS Negl Trop Dis. 2025 May 13;19(5):e0013083. doi: 10.1371/journal.pntd.0013083 (PMC12119002; doi:10.1371/journal.pntd.0013083)
Supplement: S1 Table — (DOCX) [file pntd.0013083.s003.docx]

|  | | **Total**  **n/N (%)** | **Epilepsy**  **n/N (%)** | **No epilepsy**  **n/N (%)** | **p-value** |
| --- | --- | --- | --- | --- | --- |
| Detailed serological results^£^ | Both tests negative | 5686/6288 (90.4%) | 375/433 (86.6%) | 5310/5855 (90.7%) | <0.001 |
|  | At least one test positive | 602/6288 (9.6%) | 58/433 (13.4%) | 544/5855 (9.3%) |  |
|  | Both tests positive | 58/6288 (0.9%) | 14/433 (3.2%) | 44/5855 (0.8%) |  |
|  | ELISA positive only | 283/6288 (4.5%) | 27/433 (6.2%) | 256/5855 (4.4%) |  |
|  | Immunoblot positive only | 261/6288 (4.1%) | 17/433 (3.9%) | 244/5855 (4.2%) |  |

S1 Table. Comparison of serological results and epilepsy screening between different groups.
