# Supplementary material for: Cysticercosis and neurocysticercosis in people from Mocuba district, Zambézia province: A Mozambican community-based study
Source: PLoS Negl Trop Dis. 2025 May 13;19(5):e0013083. doi: 10.1371/journal.pntd.0013083 (PMC12119002; doi:10.1371/journal.pntd.0013083)
Supplement: S2 Table — (DOCX) [file pntd.0013083.s004.docx]

S2 Table. Neurocysticercosis prevalence: observed, extrapolated, and post-stratified estimates.

|  |  | **NCC** | **Number** | **Prevalence** |
| --- | --- | --- | --- | --- |
| General population  (n=6295) | Observed | Any type of NCC | 9 |  |
|  |  | Active stage NCC | 2/9 |  |
|  | Extrapolated | Any type of NCC | 126 | 2.0% |
|  |  | Active stage NCC | 24 | 0.4% |
| Screened positive for epileptic seizures  (n=433) | Observed | Any type of NCC | 8 |  |
|  |  | Active stage NCC | 2/8 |  |
|  | Extrapolated | Any type of NCC | 26 | 6.1% |
|  |  | Active stage NCC | 7 | 1.6% |
